# Bridging the Precision Gap in Rheumatoid Arthritis: Spatial Transcriptomics, Spatial Proteomics, and Artificial Intelligence in Precision Health

**DOI:** 10.3390/biomedicines14030668

**Published:** 2026-03-14

**Authors:** Maliha Mashkoor, Shihua Zhang, Allan Stensballe

**Affiliations:** 1 Department of Health Science and Technology, The Faculty of Medicine, Aalborg University, 9000 Aalborg, Denmark; maliham@hst.aau.dk; 2 Sino-Danish Center for Education and Research, University of Chinese Academy of Sciences, Beijing 100190, China; zsh@amss.ac.cn; 3 State Key Laboratory of Mathematical Sciences, Academy of Mathematics and Systems Science, Chinese Academy of Sciences, Beijing 100190, China; 4 School of Mathematical Sciences, University of Chinese Academy of Sciences, Beijing 100049, China; 5 Key Laboratory of Systems Health Science of Zhejiang Province, School of Life Science, Hangzhou Institute for Advanced Study, University of Chinese Academy of Sciences, Chinese Academy of Sciences, Hangzhou 310024, China; 6 Clinical Cancer Research Center, Aalborg University Hospital, 9000 Aalborg, Denmark

**Keywords:** rheumatoid arthritis, machine learning, artificial intelligence, large language models, multiomics, spatial omics, transcriptomics, proteomics

## Abstract

Rheumatoid arthritis (RA) is a chronic autoimmune disease characterized by complex immune cell associations and continuous joint damage. Personalized clinical assessment and treatment options for RA remain hindered by a precision gap due to an inability to precisely match current global treatment strategies to individual molecular and spatial disease profiles. Recent advances in spatial transcriptomics and proteomics offer unprecedented opportunities to map molecular heterogeneity and spatial heterogeneity within RA tissues by identifying immune microenvironments activated during the disease, thus enabling precise therapeutic targeting. These techniques address the precision gap in RA by identifying distinct pathogenic subpopulations and cellular niches, providing insights into the biomolecules that possess significant therapeutic responses and are involved in disease progression. This review synthesizes recent findings demonstrating how spatial omics technologies, including spatial transcriptomics and proteomics, together with artificial intelligence, are transforming precision rheumatology.

## 1. Introduction: Insights into Rheumatoid Arthritis Pathogenesis

In rheumatology, the complex and elusive pathophysiology in diseases such as rheumatoid arthritis, systemic lupus erythematosus, and Sjögren’s syndrome remains a challenge for personalized treatment using single biomarkers. RA is a chronic inflammatory autoimmune disease that primarily affects joint function by inducing inflammation, chronic pain, and swelling, which eventually leads to joint destruction and functional impairment [1]. The disease is incurable and can directly affect quality of life; therefore, the early detection of RA is significant in substantially preventing joint deformity [2].

The pathogenesis of RA is complex and not completely understood. However, in general, activated immune cells recognize epitopes in the synovium via sophisticated immunological mechanisms, which stimulate localized inflammation [3]. RA onset has three main stages, namely the preclinical stage, the early RA stage, and the established RA stage. In the preclinical RA stage, anti-citrullinated protein antibodies (ACPAs) in the synovium are generated in response to different citrullinated neoantigens and modified self-proteins, resulting in a subclinical inflamed synovium. Citrullinated neoantigens presented by MHC class II molecules activate CD4+ T cells and thereby initiate adaptive immune responses. These activated T cells subsequently promote B-cell differentiation and autoantibody production, including anti-citrullinated protein antibodies (ACPAs) and rheumatoid factor (RF), while also contributing to the release of chemokines and proinflammatory cytokines.

Swelling and chronic pain in RA’s early stage are significant hallmarks of active inflammatory processes. The early RA stage mainly encompasses the first 6 months from the onset of the initial symptoms. In the early stage, synovial inflammation is mainly dominated by the infiltration of CD4+ T cells, monocytes, fibroblasts (FLS), and macrophages in the synovial membranes [4]. In this stage, ACPA production, along with the generation of other autoantibodies, including RF, is amplified [3]. Moreover, FLS trigger the production of different chemokines and cytokines, such as interleukin (IL) or tumor necrosis factor (TNF), macrophage migration inhibitory factor (MIF), and IL-17. IL-17 activates bone resorption, which eventually leads to the generation of synovial fluid.

The early RA stage is further followed by the formation of rheumatoid pannus and increased immune cell accumulation in the synovium in established RA [3,5]. The pannus grows further, produces excessive inflammatory cells, and invades the surrounding cartilage and joints during established RA, eventually leading to local matrix degradation [6]. As this pannus is immunologically highly aggressive, it is important to provide timely medical attention to patients; otherwise, these cells will invade adjacent structures, leading to joint fusion and progressive erosion, which eventually further stimulate deformities [7].

ACPAs trigger the flow of different inflammatory cells and vascular permeability to the synovium and hence make RA a highly heterogeneous disease [2,3]. ACPAs have significant diagnostic and prognostic value in RA, and they are strongly associated with the triggering of clinical symptoms, extra-articular clinical manifestations, disease progression, and sensitivity to therapies [3,8,9]. Histologically, rheumatoid pannus is the main hallmark of RA and is regarded as the ‘window of opportunity’, because it embodies a heterogeneous inflammatory cell infiltrate of activated immune cells [10].

RA treatment options range from medications to physical and occupational therapy and sometimes surgery. Previously, RA patients were mainly treated with different types of early DMARDs, such as glucocorticoids or NSAIDs. However, now, they are mainly treated with different types of disease-modifying anti-rheumatic drugs (DMARDs), such as conventional synthetic (cs) DMARDs or biologic (b) DMARDs. Among csDMARDs, methotrexate is the most commonly used therapeutic approach for the treatment of RA, followed by TNF inhibitors from the group of bDMARDs [11].

Advances in high-throughput technologies and artificial intelligence have led to major breakthroughs in precision and personalized medicine over the past decade. These technologies have evolved to resolve challenges in the domains of patient stratification and personalized treatment management. They have also made significant contributions to biomarker discovery, aiding in clinical decision-making, enhancing diagnosis, and refining disease prognosis [12]. Although these approaches have addressed therapeutic challenges for several diseases, this is an ongoing goal for RA, where their full potential has not yet been utilized due to the disease’s complex nature.

This review examines the recent developments in the field of spatial transcriptomics (ST) and proteomics in RA, while highlighting the limitations and challenges posed by spatially resolved approaches. We highlight and discuss how these approaches bridge the precision gap by elucidating spatial disease heterogeneity, predicting the therapeutic response, and revealing new biomarker landscapes. Furthermore, we discuss how artificial intelligence (AI) and machine learning (ML) frameworks can be used for spatial omics data to propel a new era of spatially informed precision rheumatology.

## 2. Spatial Transcriptomics (ST): Overview of Different Frameworks

In contrast to bulk RNA-seq, which averages gene expression across different cell populations along with masking cellular heterogeneity, and scRNA-seq, which disrupts spatial contexts through tissue dissociation, ST enables gene expression profiling within an intact tissue architecture, thus preserving the spatial context [13]. The technology mainly employs next-generation RNA sequencing, while considering the spatial information at a specific tissue locus, which can be further preserved and mapped back to its original histological locations [14].

Based on the platform, ST technologies are classified into two main types, i.e., sequencing-based ST (sST) technologies and imaging-based ST (iST) technologies [15]. sST technologies use spatial barcoded beads that capture information related to the locations of specific regions of interest (ROI) and are divided into two types, namely in situ barcoding/spatial capturing technology and microdissection-based methods.

In situ barcoding revolves around capturing the entire transcriptome spatially at barcoded spots in situ by using oligo DNA probes, which are then sequenced ex situ [16]. The microdissection-based method considers specific regions of interest (ROI) to obtain the location of the targeted transcript via microfluidics or microdissection on a chip, which is then further sequenced to quantify gene expression [17].

iST utilizes hybridization and fluorescence-based techniques to characterize the genetic expression in an intact tissue architecture. It has two sub-branches, namely in situ hybridization (ISH) and in situ sequencing (ISS) [18,19]. The ISS technique captures and sequences RNA in its tissue context using sequencing by ligation (SBL), while ISH qualitatively profiles the gene expression patterns in tissue sections by imaging complementary sequences with fluorescently labeled probes [20].

iST typically features a higher resolution while offering higher sensitivity and specificity as compared to sST technologies, which incorporate unbiased transcriptome coverage [21,22]. Additionally, iST-based methods can capture transcripts at the single-cell level, but, despite offering an enhanced spatial resolution as compared to sST platforms, iST-based methods are dependent on advanced imaging equipment and data processing frameworks and are confined to selected target panels [23,24].

**Figure 1:**
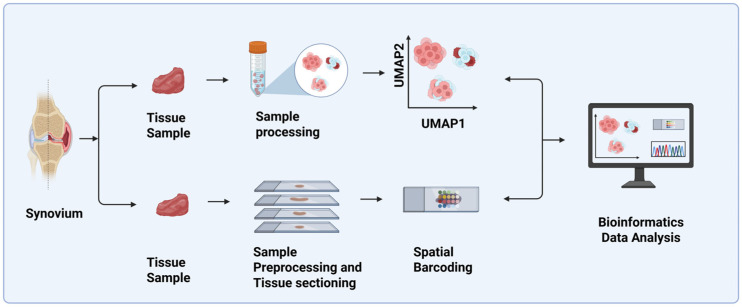
Schematic diagram of the basic workflow of spatial transcriptomics and scRNA-seq analysis. Tissue samples are extracted from the synovium and then subjected to sample preprocessing and tissue sectioning. This is further followed by spatial barcoding, integration with scRNA data, and bioinformatics data analysis. Created in BioRender. AAU, P. (2026) https://BioRender.com/imr12i1 (accessed on 8 March 2026).

ST is often used together with other technologies such as single-cell sequencing (scRNA-seq) to characterize and quantify the gene expression and cellular interactions in a cell at a particular locus within a specific tissue architecture (Figure 1). This combination tends to strengthen the multimodal spatial understanding that is lost during cell dissociation in scRNA-seq. The integration of these two techniques facilitates the discovery of novel therapeutic and diagnostic molecular signatures for the treatment of autoimmune diseases such as RA [25].

## 3. Spatial Transcriptomics: Illuminating Disease Heterogeneity

RA is a highly dynamic and heterogeneous disease in which the synovium incorporates a wide array of molecular signatures and diverse immunological populations. These populations exhibit varied sensitivity and resistance to treatment, which limits the ability to effectively treat the disease. This heterogeneity can generally be broadly classified as inter- and intra-level heterogeneity. The heterogeneity among the patient population can be defined as inter-heterogeneity. This can be reflected in the serological status of RA patients, where seropositive RA patients maintain different immunological dominance as compared to seronegative RA patients.

**Figure 2:**
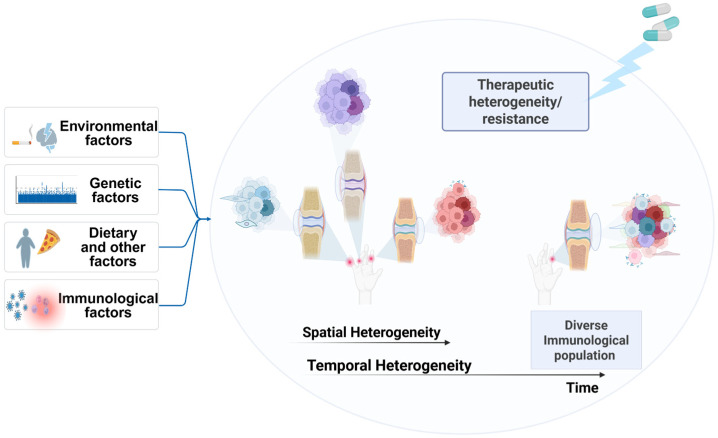
Heterogeneity exhibited in RA at different loci. Spatial heterogeneity consists of differences in immune cell populations between different loci within the same individual, while temporal heterogeneity represents heterogeneity at a specific locus over a period of time, which can lead to changes in immune cell populations. The figure also shows the risk factors contributing to RA heterogeneity, which can include environmental factors, genetic factors, immunological factors, and dietary factors. Therapeutic heterogeneity in RA can eventually lead to therapeutic resistance. Created in BioRender. AAU, P. (2026) https://BioRender.com/epmody3 (accessed on 8 March 2026).

The site-specific distributional heterogeneity within a patient at multiple affected sites can be termed intra-level heterogeneity. Intra-level heterogeneity can be further classified as spatial and temporal heterogeneity. Spatial heterogeneity is a site-dependent variation within a single individual, where a person can exhibit differences in morphological, molecular, and cellular distribution across multiple disease sites. Temporal heterogeneity can be defined as variations in the cellular or molecular characteristics of a patient at a particular locus over the course of time (Figure 2). This type of heterogeneity can arise due to multiple factors, including exposure to various disease risk factors, variations in the treatment course administered to patients, or changes in the molecular, cellular, and functional characteristics of the disease over time.

In contrast to conventional bulk sequencing and scRNA-seq, ST can capture spatially heterogeneous genetic information and can resolve microenvironmental variation at the tissue level, which the other two technologies fail to incorporate. ST can be used to investigate different cellular mechanisms, immunopathology, and diversities governing different complex autoimmune diseases, including RA, to expand the understanding of various pathophysiological processes involved in a disease at a particular locus [26]. ST can facilitate patient stratification by histologically classifying the molecular signatures embodied in the tissue architecture. This capability is particularly transformative for RA, as the disease is characterized by localized immune cell clustering, tertiary lymphoid structures, and fibroblast activation within affected joints.

## 4. Precision Gap in Rheumatoid Arthritis

RA is a spatially heterogeneous disease with varied cellular diversity and a distinct molecular composition. It has differential immune infiltration and varied tissue damage exhibited across different affected joints, leading to dispersed inflammation and spatially and temporally heterogeneous immune cell infiltration. In general, current therapeutic interventions in RA involve using ‘treat-to-target’ (T2T)-based approaches to attenuate the symptoms and to maintain remission for an extended period of time [27,28]. In this type of treatment strategy, after every 3 or 6 months, depending on the stage of RA, treatment outcomes are assessed for the administered treatment course. However, T2T fails to consider patient-specific profiles and the individualized immune signatures activated in the patient, as, during the assessment period, if the medication is unable to achieve the targeted outcomes, the treatment is modified without considering the patient’s disease profile [29].

**Figure 3:**
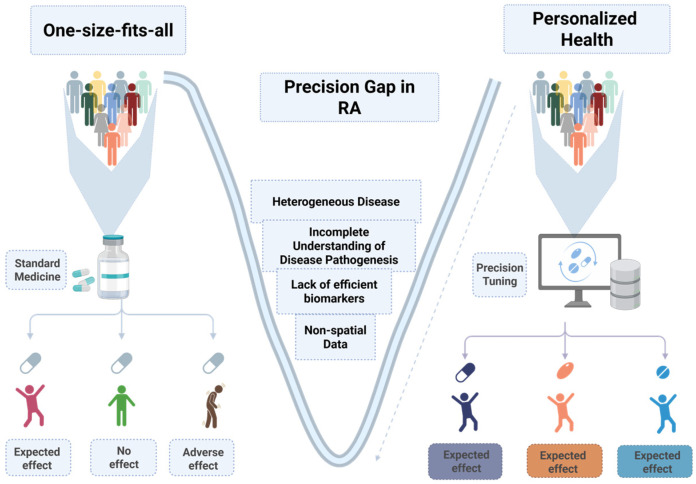
The precision gap in rheumatoid arthritis arises from a limited understanding of the disease’s spatial heterogeneity, impeding individualized therapy; a lack of effective biomarkers; and non-spatial data. Spatial transcriptomics, spatial proteomics, and AI-driven integration collectively bridge this gap by enabling molecular and spatially informed personalized treatment strategies. Created in BioRender. AAU, P. (2026) https://BioRender.com/gsu0lgw (accessed on 8 March 2026).

Despite substantial therapeutic advances in other diseases, RA clinical management remains largely uniform, leading to considerably variable therapeutic responses and incomplete remission. This challenge in RA can be termed a precision gap, which reflects our limited ability to consider the personalized immunological landscape, predict therapeutic outcomes, and stratify patients based on their molecular signatures. The gap is further extended due to an incomplete understanding of the disease’s pathogenesis, insufficient biomarker validation, and the absence of integrative diagnostic and clinical frameworks that can account for the spatial and molecular complexity of the patient profile (Figure 3).

Currently, two primary and persistent knowledge gaps substantially contribute to the present limitations caused by the precision gap in RA. Firstly, this gap mainly stems from the limited understanding of the disease’s pathogenesis. Although scientists have achieved progress in characterizing biomarkers and the pathobiology, there are still several domains that lack clarity and a comprehensive understanding (Figure 3). The scarcity of sufficient diagnostic, prognostic, and therapeutic disease biomarkers that reflect disease onset, progression, and responses further complicates efforts directed towards precision medicine. Secondly, another major driver of the precision gap is the inability to effectively predict disease outcomes. These issues together hinder efforts to implement precision tuning and pose challenges in therapeutic decision-making and, eventually, in achieving optimal patient outcomes.

Bridging the precision gap through the incorporation of spatial biology offers the most promising path towards personalized therapy for RA patients. The most significant aspect in this case is to gain a deeper understanding of the pathogenesis of RA and to predict remission, relapse, and other outcomes associated with the disease. This can be accomplished by leveraging advanced spatial omics platforms with the goal of obtaining insight into the positional and functional contexts of gene expression, tissue morphologies, and the distribution of immunological infiltration in the tissue microenvironment.

## 5. Bridging the Precision Gap in the Pathogenesis of Rheumatoid Arthritis Through Spatial Transcriptomics (ST)

Spatial transcriptomics (ST) technologies characterize gene expression in tissues while preserving spatial location information. Recent studies have demonstrated the potential of ST to uncover spatially resolved immune landscapes that define disease heterogeneity in seropositive and seronegative RA patients. For example, Ståhl et al. [13] employed spatial transcriptomics with RNA-seq analysis to investigate the spatial variability in synovial tissue interactions at tertiary lymphoid organ (TLO) inflammation sites in seropositive and seronegative RA patients. In seropositive RA patients, signals for genes related to cell chemotaxis, leukocyte migration, and humoral immune responses were observed, whereas seronegative RA patients showed oxidative stress and receptor-mediated endocytosis gene signals within inflamed sites. By spatially profiling RA synovium biopsies, they primarily emphasized the role of leukocytes in RA tertiary lymphoid organs (TLOs). Furthermore, high expression of CXCL13 and CXCL12/CCL19 has been reported in seropositive patients compared with seronegative patients. Seropositive patients have also been reported to have smaller proportions of DCs compared with seronegative patients [27].

Another study using scRNA-seq and spatial transcriptomics in ACPA+ and ACPA− patients reported small, localized, and distinct B-cell leukocyte subsets in early RA patients within two days of diagnosis, before treatment initiation. The study also reported an abundance of memory cells and plasma cell niches in the early inflamed synovium, with these cells showing high CXCR4 expression and strong CXCL12 expression in the surrounding inflamed tissues. Furthermore, the study suggested that plasma cell and B-cell differentiation are independent of ACPA status, contrary to earlier speculation that only ACPA+ RF patients have higher concentrations of B/T cells due to autoantibody abundance. Although the study provides further insight into RA’s pathogenesis, its findings cannot be generalized to a broader population and it is limited by the sample size, encompassing only four samples, with two in each of the ACPA+ and ACPA- RA patient groups [28].

Kenney et al. used ST and scRNA-seq to provide additional insights into lymphatic dysfunction and cellular dysregulation in RA in the tumor necrosis factor transgenic (TNF-Tg) mouse model [30]. The results showed upregulated expression of IgG2b+ plasma cells and downregulated expression of Fth1, along with ALCAM+ macrophages and CD6+ T cells responsible for IgG2b class-switching in more advanced RA [29]. Cutaneous vasculitis consists of small skin lesions that are formed in autoimmune diseases such as RA, and they can trigger premature mortality. In an attempt to attain deeper insight into the gene expression and pathogenesis involved in vasculitis, Tsujii et al. used single-cell spatial transcriptomics, where they reported that RA vasculitis patients demonstrated high expression of the MMP1 gene in fibroblasts [31].

ST and scRNA-seq technologies have been used to explore the inflammatory and immune-related cellular impairments and communication that trigger a specific pathophysiology and can lead to differential and ineffective therapeutic resistance in fibroblasts. Smith et al. [32] used single-cell sequencing, multiplexed imaging, and spatial transcriptomics to identify the drivers of cytokine signaling in inflamed synovial tissue in RA. They identified leukocyte-based cytokines, including tumor necrosis factor (TNF), interferon (IFN)-γ, and IL-1β, as the factors responsible for transcriptional heterogeneity, as well as demonstrating the formation of four different FLS states in the inflamed synovium.

Together, ST and scRNA-seq can expand our understanding of the dominant cell types and complex molecular and aberrant inflammatory pathways activated during the pathogenesis of a disease. Fibroblast activation is further explored through monitoring fibroblast activation protein (FAP) during inflammation using positron emission tomography, scRNA-seq, and ST. A study has reported the downregulation of FAP, which is suspected to be caused by the substitution of pro-inflammatory MMP3+/IL6+ fibroblasts (high FAP internalization) for CD200+DKK3+ fibroblasts (low FAP internalization). CD200+DKK3+ fibroblasts further stimulate the induction and stability of type 2 innate lymphoid cells and the CD200-CD200R1 signaling pathway in the mesenchymal compartment [33].

In addition, a study explored the polarization states and distribution of macrophages in RA vs. OA using the combination of ST and scRNA-seq. The authors identified three groups of macrophages in the synovium, namely M0-like MARCO+ Mϕ1, M2-like CSF1R+ Mϕ2, and M1-like PLAUR+ Mϕ3, which showed differential distributions. OA patients showed the upregulation of CXCL2, CXCL1, IL1B, TNFAIP3, ICAM1, CXCL3, PLAU, CCL4L2, CCL4, and TNF in the NF-kappa B signaling pathway in the specified macrophages, while HOXB6, STAT1, and NFKB2 were highly expressed in the macrophages in RA tissues. These biomarkers can be further assessed as potential therapeutic targets [34].

To further explore the immunological and pathological differences between RA and other diseases, Carlberg et al. [35] used spatial transcriptomics on RA and SpA synovial biopsies and reported differential pathological mechanisms between the two diseases. They suggested that SpA had stronger mesenchymal cell signatures and more cartilage-related genes as compared to RA, while B- and T-cell-related genes were found in high proportions in both diseases.

The intersection of scRNA-seq and ST also provides critical insights into cell–cell interactions, cell–ligand interactions, differentially regulated pathways, the tissue microenvironment, and discrete cell populations within a tissue. Lai et al. [36] used single-cell data with spatial transcriptomics to characterize the underlying mechanisms and cell–cell interactions governing chemokine signaling in RA patients. They identified the crucial role of CD8+ T cells and endothelial cells (ECs) and the potential involvement of the ligand–receptor pair CCL5-ACKR1 in chemokine RA signaling, where these immune-related components stimulate RA progression by facilitating immune cell translocation into the RA synovium.

Another recent study by Bhamidipati et al. [37] used 30 synovial biopsies collected 6 months before and after patients received RA treatment. The main purpose of the study was to identify the molecular determinants of treatment resistance in RA patients. They identified the potential role of fibrogenic signaling, including TGFβ isoform expression, along with Notch signaling, which could be responsible for treatment resistance in RA patients. Additionally, Romoff et al. [38] used spatial transcriptomics to further identify the immunomodulatory role of FLS in the inflamed synovium. They further characterized the therapeutic roles of ID01 and CD69 within RA lymphoid structures and proposed the immunosuppressive effects of FLS.

## 6. Spatially Informed Outcome Measures and Flare Prediction

RA is significantly affected by the precision gap. This gap substantially impedes the effective stratification of patients based on their unique clinical and molecular profiles. Consequently, this leads to challenges in the prediction of the disease course and in the identification of individuals who could respond to a specific treatment, as treatment options are instead administered uniformly. Therefore, the inability to match suitable treatments to patients at the appropriate time continues to exacerbate the precision gap in clinical care, thus limiting the potential of precision medicine to improve outcomes and minimize superfluous interventions.

Rheumatic patients in remission can experience disease flares, which represent a fluctuating disease course with adverse disease activity. Depending on the duration of the flare, its intensity, and its persistence, it can pose challenges in optimizing disease outcomes and can lead to either a change in current therapy or the initiation of a different treatment course, which can be time-consuming if the disease is progressing swiftly. For the management of RA, it is important to discover biomarkers that can be used to predict the frequency, occurrence, and severity of RA flares. One such effort used ST to find biomarkers predictive of RA flares after treatment adjustment. The results revealed the CD68pos macrophage pattern as a biological signature in the synovial tissue that could be used to differentiate between remission patients and patients at risk of RA flares [39].

A study combining scRNA-seq analysis and ST identified the differential expression of DCs in patients who had attained remission and those who experienced flares. The authors noted that, prior to having flares, patients who had attained remission were found to have ST-DC3 clusters. These clusters can be used as biomarkers to predict the occurrence of RA flares and to restore immune tolerance in RA patients. They also reported the wide dispersion of AXL+ DC2 populations in active RA patients, which was later absent in the remission stage and can possibly lead to the development of RA flares. Moreover, they reported that heterogeneous inflammatory DC3 clusters in the synovium mainly dominated the hyperplastic lining in activated RA, while the populations of CCR7+ DC2s in lymphoid aggregates were found in later RA stages [40].

The mechanisms involved in relapse in RA have not been fully investigated; therefore, Meng et al. [41] attempted to assess its transcriptional heterogeneity and identify the mechanisms involved in RA relapse by examining in fibroblast-like synoviocytes (FLSs) using scRNA/ST-seq. They reported the potential role of fibroblast growth factor (FGF) from the FLS lining in RA relapse. They also proposed the involvement of the FGF receptor (FGFR) 1 inhibitor in reducing the effects of bone erosion; meanwhile, the FGF10 protein showed involvement in exacerbating bone erosion. The study proposed the suppression of FGF10/FGFR1 in the FLS lining as a potential therapeutic approach that could be explored for the treatment of RA relapse.

## 7. Facilitating Precision Tuning by Employing ST

Tailoring therapeutic strategies by mapping the diversity of information encoded in the patient profile with the therapeutic profile of the drug can be termed precision tuning or therapeutic matchmaking. Meanwhile, the safety profile of the drug, variations in drug effectiveness, vulnerability to side effects, the consideration of comorbidities, the assessment of likely responses to treatment, and the prediction of the adverse outcomes also play a crucial role in precision tuning. In the context of RA, among these factors, patient heterogeneity also plays a significant role, influencing the implementation of personalized medicine approaches.

Current advancements in ST and scRNA-seq technologies have provided unprecedented insights into the tissue microenvironment and drug resistance. Zheng et al. [42] used these technologies and found ITGA5+ fibroblasts to be involved in RA DMARD drug resistance, employing a transcriptome unit comprising POSTN, COL3A1, CCL5, and TGFB1. This transcriptome unit can help in overcoming resistance to DMARDs and thus can be used as a suitable therapeutic target. They also profiled the progression and pathogenesis of RA in the synovium tissue, where they found ITGA5+ synovial fibroblasts to be facilitators of RA progression during the early stages by remodeling the pro-inflammatory tissue microenvironment.

In RA, the diversity of the molecular and cellular populations harbored in a patient allows the cells to evolve, evade the effects of the therapy, and then eventually become resistant to its therapeutic effects. Thus, patient heterogeneity not only determines differential treatment outcomes but also allows the cells to adapt to and escape therapeutic interventions. As a result, the disease progresses further, and the desired treatment outcomes are not achieved despite consistent efforts using conventional therapies. This highlights the significance of characterizing the heterogeneity and genetic diversity of the localized disease microenvironment to design effective treatment strategies.

In this context, a study was conducted by Rivellese et al. [43] in which they employed ST to identify the microenvironment, spatial dependencies, and intercellular communication governing the drug response in different synovial tissue pathotypes. The study aimed at identifying pathways that were activated during resistance to rituximab and tocilizumab. They used RNA-seq together with ST to examine the synovium biopsies of 164 patients to identify differences between responders and refractory patients. The main aim of the study was to facilitate therapeutic matchmaking by integrating transcriptomic and spatial analysis to effectively stratify patients into groups that respond to a particular therapeutic intervention based on synovial heterogeneity. They reported that myeloid-rich phenotypes respond better to tocilizumab, and rituximab’s efficacy is greater in cases of a lymphoid-dominated synovium. In contrast, synovia with high proportions of macrophages and fibroblasts, along with low proportions of lymphocytes, exhibited high resistance to both medications. The study also noted the expression of pro-fibrotic and inflammatory mediators in stromal-dominant areas, even after therapeutic interventions.

## 8. Spatial Proteomics: Completing the Functional Map

Spatial proteomics extends the spatial omics paradigm by quantifying protein expression and localization directly within the tissue context. Because proteins are the functional effectors of cellular signaling, integrating proteomic data with ST contributes to a more complete understanding of disease mechanisms. This is especially critical in RA, where post-transcriptional modifications, cytokine signaling, and extracellular matrix remodeling shape disease activity and therapeutic responses [44].

Recently, spatial proteomics has emerged as a powerful technique to understand tissue complexity. It enables the interpretation of high-quality images of the spatial composition of protein expression within a tissue [45]. Additionally, the technique can be combined with other approaches, such as biomedical imaging analysis, machine learning, and scRNA-seq, to obtain further insights into disease pathogenesis [46]. These proteomic advancements could help to alleviate the mismatch between treat-to-target strategies and spatially heterogeneous synovial biology.

Spatial proteomics is divided into two types: fluorescence-based antibody spatial proteomics and mass spectrometry (MS)-based spatial proteomics. In fluorescence-based antibody spatial proteomics, to identify the exact location of a protein, fluorescently labeled probes or antibodies are employed to determine the spatial distribution of proteins [47]. MS-based spatial proteomics includes LC-MS-based spatial proteomics and mass spectrometry imaging (MSI) [48]. MSI is used to obtain molecular mass and location information in the tissue by integrating spatial positioning information with mass spectrometry. LC-MS-based spatial proteomics approaches allow for the direct detection of proteins in tissue sections by using liquid chromatography with tandem mass spectrometry [49].

Recent studies, such as those by Wang et al. [50], have shown that combining spatial proteomics (from laser capture microdissection (LCM) coupled with mass spectrometry-based proteomics) with scRNA-seq data can reveal cell types enriched in pathogenic protein networks, thus bridging the gap between transcriptional changes and functional outcomes. The study characterized the varied expression of different membrane proteins (TYROBP, AOC3, SLC16A3, TCIRG1, and NCEH1) and extracellular matrix (ECM) proteins (PLOD2, OGN, and LUM) in the pathophysiology of the synovium. They further enriched these proteomics-based data with scRNA-seq data to identify cellular regions that were enriched in these proteins. The different cell types included T cells, fibroblasts, NK cells, myeloid cells, B cells, and synovial endothelial cells. They proposed the use of the identified proteins as potential therapeutic targets.

Another study by Lima et al. [51] used spatial proteomics to identify the molecular network within fibroblasts that is associated with the response to csDMARDs and remission within RA. They found the LYVE1^+^CD206^+^ tissue-resident macrophage network to be disturbed during activated RA. However, they suggested its stability after treatment with csDMARDs. They also identified the roles of chemokines, annexins, and TAM receptors (TYRO3, AXL, MERTK) in the regulation of the specified macrophage network.

## 9. Limitations in the Clinical Translation of the Precision Gap

**Figure 4:**
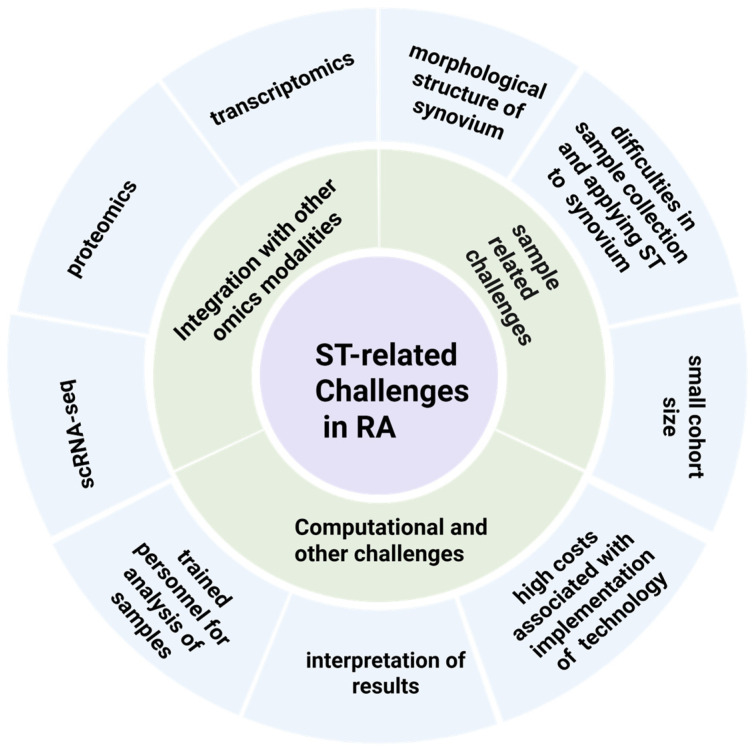
Challenges encountered by ST in RA, which mainly include those related to sample collection, integration with other omics modalities, and several other computational challenges. Created in BioRender. AAU, P. (2026) https://BioRender.com/so269jg (accessed on 8 March 2026).

As RA is a highly heterogeneous disease, precision medicine approaches play a fundamental role in its treatment and management. Precision medicine in RA leverages the information obtained from different diagnostic tests and tissue biopsies to harness the power of big data and to allow for more tailored therapeutic approaches. In many areas, such as rheumatology and oncology, precision medicine represents a vital pillar in the development of effective therapies that utilize the molecular profiles of patients. However, precision medicine approaches for RA are not routinely practiced in the clinic, and the current treatment options largely rely on treat-to-target strategies [52]. This hinders the development of focused treatment strategies and precise disease-specific targeting, which in turn widens the precision gap in RA clinical care (Figure 4).

Despite the availability of different spatial transcriptomics approaches, the high costs associated with their implementation present difficulties in the adoption of these technologies on a wider scale. The generation and interpretation of the results are dependent on trained personnel, and many laboratories lack sufficient resources to address this challenge. Commercial tools are available for control, standardization, and data analysis, but they can be limited by rigid scalability and are often economically unfeasible for individual laboratories, while technical variations and workflow bottlenecks can affect the tools’ interoperability and the results. The data generated from spatial technologies, especially imaging data, can be regarded as big data and may need specialized, allocated storage units and high computational power.

In RA, ST is also hindered by small cohort sizes and the limited numbers of samples collected from RA patients. This is mainly because the synovium is a delicate, gelatinous, and soft structure with a loose connective matrix and a complex heterogeneous tissue architecture, posing constraints in ensuring the specific morphology and thickness needed during tissue re-sectioning for ST. This limits the ability to gain further insights into the disease. Additionally, small sample sizes further lower confidence in the evidence and limit the generalizability of the results obtained to larger populations.

Another limitation of ST is that it sometimes has to be combined with other omics technologies—mainly with scRNA-seq and RNA-seq—to further increase the resolution and enable inference about the tissue architecture. One of the key future directions for ST is its harmonization with multiomics modalities to construct molecular maps and atlases of RA’s pathology. For example, Metousis et al. integrated the use of spatial transcriptomics and spatial proteomics to construct a molecular atlas and examine the pathogenesis of high-grade serous carcinoma (HGSC) and to define novel therapeutic targets [53]. However, this can be a complex endeavor due to variations in data collection, formats, storage, and dimensionality across the two spatial omics technologies.

In heterogeneous tissues like the RA synovium, the discordance between transcriptomics and proteomics is high, and transcriptomic analysis is not always sufficient in determining the final protein expression profile. Therefore, the integration of spatial omics-based approaches is important in ensuring the characterization of post-translational disease mechanisms and in identifying patient-specific pathotypes. Moreover, bringing spatial omics into clinical rheumatology requires bridging discovery science with diagnostic and therapeutic practice. Future research should prioritize standardized sample processing, integrative bioinformatics pipelines, and the validation of spatial biomarkers across large patient cohorts. For example, one such effort by Tran et al. combined spatial transcriptomics with spatial proteomics on the same tissue sections, while using consistent analysis to identify the tumor microenvironment’s heterogeneity, across lung cancer samples at two different molecular levels [54].

## 10. Future Directions and Use of Artificial Intelligence in ST to Address Its Limitations

Artificial intelligence (AI) and machine learning (ML) have become essential in addressing the complexity of rheumatoid arthritis (RA) through the analysis of multi-layered, high-dimensional data derived from spatial transcriptomics, spatial proteomics, single-cell sequencing, and clinical imaging. Initially, most spatial analyses focused on a single omics layer, but current AI frameworks increasingly integrate several modalities, enabling the joint modeling of gene expression, protein abundance, tissue morphologies, and clinical metadata. Deep learning methods can process these rich datasets, uncover hidden biological patterns, and provide predictive models for disease activity, flare risks, and therapeutic outcomes [55,56,57,58,59,60].

Different serologic biomarkers, such as RF and ACPAs, alone are unable to fully account for RA’s heterogeneity; therefore, the discovery of additional biomarkers is required by combining spatial omics with AI to close the clinical serologic gap in RA [61]. Additionally, evaluating treatment responses to different therapeutic options and stratifying patients accordingly is vital in providing the best treatment options. For example, Bai et al. used univariate analysis in evaluating the treatment efficiency of a TNF inhibitor and then stratified patients accordingly [62]. Combining AI with ST can help in evaluating treatment resistance in clinical settings to different therapeutic options, such as TNF and JAK inhibitors; in the identification of cellular heterogeneity at a particular locus; and in developing predictive models that can refine the therapeutic options and identify the underlying, complex associations among novel disease-associated biomarkers involved in RA.

The convergence of spatial omics and artificial intelligence represents the next frontier in precision medicine, particularly in rheumatology. As RA is a complex, heterogeneous disease, combining spatial transcriptomics with spatial proteomics has great potential in understanding the synovium’s diversity [39]. However, as both transcriptomics and proteomics datasets grow in dimensionality and complexity, their integration requires advanced computational frameworks. Artificial intelligence has emerged as a vital partner in decoding these multimodal landscapes in research and clinical diagnostic support systems. AI-driven algorithms can now analyze and integrate vast, high-dimensional datasets from spatial transcriptomics and spatial proteomics studies with clinical metadata to identify disease patterns that are invisible to humans [55,56].

Spatial omics, along with artificial intelligence, can also facilitate patient stratification by histologically classifying molecular signatures and mapping the cellular neighborhood embodied in the tissue architecture. Current diagnostic, prognostic, and therapeutic approaches are unable to stratify patients based on tissue heterogeneity and cellular dynamics, as they mainly average gene expression across tissues, making it challenging to capture tissue heterogeneity. Spatial profiling, using spatial technologies along with AI, can obtain information about cellular dynamics at a particular locus by extracting spatial niches and heterogeneity at a spatial locus, thus helping in refining the therapeutic options for patients in clinical settings.

Graph-based models and image-aware architectures are particularly well suited to spatial omics. Graph neural networks (GNNs) and related methods represent each spot or cell as a node connected to its spatial neighbors, allowing the inference of cellular neighborhoods, ligand–receptor circuits, and spatial domains. Tools such as STAligner integrate multiple spatial transcriptomics slices across patients, conditions, and experimental batches by combining graph attention networks with triplet loss, thereby overcoming technical variations and spatial heterogeneity [63,64]. Methods such as STAMapper further combine spatial coordinates, gene expression patterns, and single-cell transcriptomics with a heterogeneous graph neural network to delineate high-resolution spatial domains and map cell types within tissues [65,66].

Through neural architectures such as convolutional neural networks (CNNs) and GNNs, AI can help in identifying tissue structures, characterizing gene modules, and interpreting spatial domain information from the embedding space obtained from the GNN [65]. STAGATE combines the patterns from spatial and gene expression profiles into a graph attention autoencoder framework to characterize the spatial domains found in low-dimensional latent embeddings [66]. Employing such approaches in RA allows the identification of tissue morphologies within the synovium and the prediction of key disease-driving interactions between fibroblasts, macrophages, and lymphocytes [57,59,60].

Advancing precision diagnostics by using AI models in rheumatology also bridges the gap between molecular and histopathological image data by linking spatial cellular granularity and transcriptomic signatures to digital pathology images [59,67]. For example, Ying Wu et al. [68] exploited the potential of deep learning to examine ST histology images in STATSCAN to interconnect the morphological and genetic information present in them. They identified various cell types present across the tissue sections sliced in ST, thus facilitating the resolution of cell distribution maps in uncharted areas captured in histological images.

Recent years have also witnessed a transformative rise in the use of large language models (LLMs) as tools for rheumatic research [69,70,71]. Domain-specific LLMs such as BioGPT, PubMedBERT, and GPT-4/5-based frameworks are capable of understanding and generating scientific text with contextual awareness [72,73]. In the context of RA, these models can extract and synthesize knowledge from millions of scientific publications, clinical trial reports, and electronic health records, thereby accelerating hypothesis generation and biomarker discovery. LLMs can semantically link molecular pathways, immune cell types, and clinical outcomes, enabling the construction of knowledge graphs that unify data from transcriptomics, proteomics, and imaging modalities [74,75].

However, the true transformative potential of these spatial omics approaches lies in their integration with emerging artificial intelligence and large language models (LLMs). AI enables the extraction of high-dimensional spatial patterns and the prediction of patient outcomes, while LLMs provide contextual understanding by linking molecular findings with clinical narratives and published evidence. Together, these technologies enable a continuous, learning-based precision health ecosystem where spatial omics data inform model predictions, clinical feedback refines algorithms, and AI-driven analysis accelerates biomarker discovery and therapy optimization.

The integration of AI and LLMs in RA is not without challenges. Model interpretability, data privacy, and bias remain important considerations. However, emerging frameworks in federated learning and privacy-preserving computation offer solutions that allow multi-institutional model training without compromising patient confidentiality [59,72,76]. As computational infrastructure and data-sharing initiatives expand, the synergy between spatial omics and AI is expected to significantly enhance translational discovery and clinical precision in RA management.

**Figure 5:**
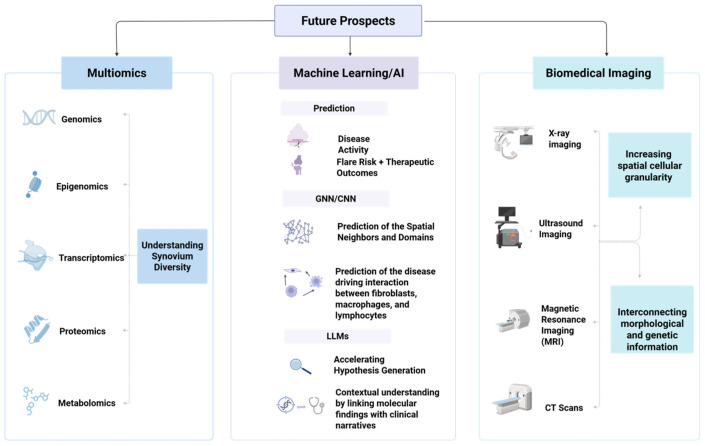
Prospects of multiomics, machine learning/AI, and biomedical imaging analysis in addressing the limitations of ST for RA. AI and machine learning can aid in various prediction-related tasks. While biomedical imaging analysis can help in integrating and enhancing the insights provided by spatial omics through segmentation analysis, combining different spatial omics modalities can provide deeper insights into synovium diversity and heterogeneity. Created in BioRender. AAU, P. (2026) https://BioRender.com/72648rn (accessed on 8 March 2026).

Looking forward, AI-augmented spatial omics will drive a paradigm shift from descriptive biology to actionable clinical insights in rheumatology, with the individual patient as the focus (Figure 5). As computational infrastructure, curated spatial omics resources, and data-sharing initiatives expand, the synergy between spatial biology and intelligent computation is expected to move rheumatology from descriptive synovial biology towards actionable clinical decision support into routine clinical care. AI-augmented single-to multi-omics integration in spatial transcriptomics and proteomics will be central to closing the precision gap in RA by helping clinicians to match the optimal therapy to each patient at the appropriate time.

## 11. Conclusions

Spatial transcriptomics and deep visual proteomics are transforming our understanding of rheumatoid arthritis by revealing the spatial logic of inflammation, tissue pathotype remodeling, and the discovery of biomarkers for events such as remission and adverse effects. These technologies directly address the precision gap that has long limited patient-specific disease management by allowing scientists to visualize disease mechanisms within their native tissue contexts. Clinically, spatial biomarkers have the potential to inform patient stratification, predict flare risks, and optimize biologic therapy selection. Integrating multiomics and AI into clinical workflows could further enable real-time data interpretation and decision support. Together, these developments signify a shift from empirical to evidence-based, spatially resolved precision medicine and precision health.

## Data Availability

No new data were created or analyzed in this study.

## Abbreviations

The following abbreviations are used in this manuscript:

RA	Rheumatoid arthritis
ST	Spatial transcriptomics
sST	Sequencing-based spatial transcriptomics
iST	Imaging-based spatial transcriptomics
scRNA-seq	Single-cell RNA sequencing
FLS	Fibroblast-like synoviocytes
FAP	Fibroblast activation protein
DMARDs	Disease-modifying antirheumatic drugs
csDMARDs	Conventional synthetic DMARDs
AI	Artificial intelligence
ML	Machine learning
LLM	Large language model
TLO	Tertiary lymphoid organ
DC	Dendritic cell
